# Aerobic Training-Induced Changes in Sedentary Time, Non-Exercise Physical Activity, and Sleep Among Breast Cancer Survivors and Postmenopausal Women Without Cancer

**DOI:** 10.3390/healthcare13192471

**Published:** 2025-09-29

**Authors:** Francesco Sartor, Leandro Ditali, Giacomo Sirtori, Teresa Morano, Federica Lancia, Alessandra Di Marco, Pascal Izzicupo, Angela Di Baldassarre, Sabina Gallina, Mirko Pesce, Simona Grossi, Antonino Grassadonia, Anastasios Vamvakis, Ines Bucci, Giorgio Napolitano, Andrea Di Blasio

**Affiliations:** 1 Department of Medicine and Ageing Sciences, “G. d’Annunzio” University of Chieti-Pescara, Via Polacchi L. 11, 66100 Chieti, Italy; francesco.sartor@unich.it (F.S.); giacomo.sirtori@phd.unich.it (G.S.); moranoteresa@gmail.com (T.M.); lanciafederica@gmail.com (F.L.); diemmealessandra@gmail.com (A.D.M.); pascal.izzicupo@unich.it (P.I.); angela.dibaldassarre@unich.it (A.D.B.); mirko.pesce@unich.it (M.P.); ines.bucci@unich.it (I.B.); giorgio.napolitano@unich.it (G.N.); andrea.diblasio@unich.it (A.D.B.); 2 School of Psychology and Sport Science, Bangor University, Bangor LL57 2DG, UK; 3 Eusoma Breast Centre, “G. Bernabeo” Hospital, ASL02 Lanciano-Vasto-Chieti, c.da S. Liberata, 66026 Ortona, Italy; simona.grossi@asl2abruzzo.it (S.G.); antonino.grassadonia@unich.it (A.G.); 4 Department of Neurosciences, Imaging and Clinical Sciences, “G. d’Annunzio” University of Chieti-Pescara, Via Polacchi L. 11, 66100 Chieti, Italy; sabina.gallina@unich.it; 5 Complex General Surgery Unit with Specialization in Breast Surgery “G. Bernabeo” Hospital, ASL02 Lanciano-Vasto-Chieti, c.da S. Liberata, 66026 Ortona, Italy; 6 Department of Innovative Technologies in Clinical Medicine and Dentistry, “G. d’Annunzio” University of Chieti-Pescara, Via dei Vestini 31, 66100 Chieti, Italy; 7 Clinical Oncology, “G. Bernabeo” Hospital, ASL02 Lanciano-Vasto-Chieti, c.da S. Liberata, 66026 Ortona, Italy; 8 Department of Nutrition and Dietetics Sciences, School of Health Sciences, Hellenic Mediterranean University, 72300 Sitia, Greece; tvamvakis@hmu.gr

**Keywords:** walking, Nordic walking, workout, breast cancer, menopause, sleep, NEPA, sedentary time, compensation

## Abstract

**Background/Objectives:** The 24 h activity cycle highlights the need to consider sedentary behavior (SED), non-exercise physical activity (NEPA), and sleep when introducing aerobic exercise. This study assessed changes in these components among breast cancer survivors (BCS) and non-oncologic menopausal women after a 3-month walking (W) or Nordic walking (NW) program. **Methods:** A total of 324 menopausal women participated: 156 non-oncologic (Meno), 102 BCS with natural menopause (BCS_Meno), and 66 BCS with medically induced menopause (BCS_Ind_Meno). Linear Mixed Effects (LME) modeling was applied. Age, BMI, hormonal therapy, cancer treatments, hypertension, sleep, and METs were included as covariates. **Results:** BCS_Meno and BCS_Ind_Meno had longer sleep durations at baseline (adj. diff.: +26.5 min/day, 95% CI 10.1 to 43.0; *p* = 0.002 and +25.7, 95% CI 6.7 to 44.6; *p* = 0.008). Sleep improved across all groups post-intervention (overall adj. effect = +17.4 min/day, 95% CI 4.8 to 30.0; *p* = 0.007). Higher sleep and METs were associated with reduced SED (sleep: β = −43.7 min/day per unit increase, −52.6 to −34.8; METs: β = −115.4, −126.4 to −104.4; both *p* < 0.001). A significant group × time interaction showed a decrease in SED in the BCS_Ind_Meno group (adj. diff. = −65.1 min/day, −102.8 to −27.4; *p* = 0.001). NEPA was negatively influenced by sleep (β = −8.7 min/day, −16.2 to −1.1, *p* = 0.024) and positively by METs (β = +121.1, 111.8 to 130.3; *p* < 0.001). NEPA increased only in BCS_Ind_Meno (adj. diff.: +70.6 min/day, 38.4 to 102.7; *p* < 0.001), not in BCS_Meno (+9.87, −18.7 to 38.4; *p* = 0.497). **Conclusions:** BCS_Ind_Meno showed the greatest benefits, with reduced SED, increased NEPA, and improved sleep. Sleep improved across all groups following aerobic interventions.

## 1. Introduction

The majority of breast cancers are hormone receptor-positive, meaning that the cancer cells express estrogen receptors (ERs), progesterone receptors (PRs), or both [1]. These tumors typically respond to hormone therapy, which either blocks the action of estrogen on cancer cells, using selective estrogen receptor modulators such as tamoxifen, or reduces estrogen production in the body through aromatase inhibitors. By targeting estrogen signaling, hormone therapy helps slow or halt tumor growth [2]. The most commonly prescribed hormone therapies for hormone receptor-positive breast cancer include aromatase inhibitors, such as exemestane and anastrozole, primarily used in postmenopausal women, and selective estrogen receptor modulators, such as tamoxifen, typically prescribed to premenopausal women. In premenopausal patients, tamoxifen is often administered alone or in combination with ovarian function suppression (e.g., gonadotropin-releasing hormone [GnRH] agonists). In certain high-risk cases, aromatase inhibitors may also be used alongside ovarian suppression. The choice of therapy depends on the patient’s menopausal status, risk of recurrence, and the potential side effect profile [3,4]. Adjuvant hormone therapy is recommended to enhance the effectiveness of primary treatments such as surgery, chemotherapy, and/or radiation [5]. It is commonly taken for five years and functions by blocking the effects of estrogen, thereby helping to prevent breast cancer recurrence [6]. According to research, adherence to hormone therapy significantly decreases the risk of breast cancer mortality and recurrence [7]. Adverse side effects associated with hormone therapy are often cited as a major reason for poor adherence [8]. Hot flushes, joint pain, fatigue, and sleep difficulties are the most common side effects [9,10], and they also contribute to a reduction in total physical activity over time, mainly due to a decrease in mild-intensity physical activity, from diagnosis up to four years later [11]. Despite the increasing trend toward physical activity and exercise, a growing body of research highlights their importance in cancer prevention, recovery, and treatment, specifically in preventing and managing many therapy-related side effects, as well as in reducing the risk of cancer recurrence [12,13].

Unfortunately, the idea that simply recommending (or engaging in) physical exercise automatically leads to reduced daily sedentary time (SED), increased non-exercise physical activity (NEPA), and improved duration and quality of sleep does not fully reflect real-life experiences. Indeed, although a substantial body of research supports that engaging in aerobic exercise improves both the quantity and quality of sleep for individuals with sleep disorders in general [14] and for breast cancer survivors (BCS) [15], this effect does not extend to SED and NEPA. Based on current research, the response of SED and NEPA to physical exercise depends on several factors, including age, sex, body fat mass, health status, baseline physical activity level, and exercise characteristics such as type, duration, intensity, frequency, and total volume [16,17,18,19,20,21,22,23]. In practical terms, women who are obese and/or have a high baseline physical activity level, and who engage in moderate-to-vigorous aerobic exercise at least three times per week for 30 to 60 min over a period of up to three months, appear more likely to decrease their NEPA and consequently increase SED, with this effect being correlated with age [16,17,18,19,20,21,22,23]: the older the individual, the greater the compensatory effect appears to be. The complexity of this phenomenon is underscored by the identification of multiple neurochemical pathways, including various mediators, that influence NEPA in both animals and humans [24], in addition to the contribution of various candidate genes and the effects of occupational and socio-cultural factors [24]. In a study of adolescent girls, the number of weekly hours spent engaging in physical activity was found to be correlated with the calcium-sensing receptor gene [25]. The leptin receptor gene has been associated with energy expenditure and physical activity in Pima Indians [26], a finding confirmed by De Moor et al. along with associations for the aromatase and the SH3-domain GRB2-like (endophilin) interacting protein 1 (SH3GL1) gene, both linked to voluntary exercise [27]; Salmén et al. further supported the aromatase association in postmenopausal women [28]. The dopamine 2 receptor gene was related to prior-year activity in women of European but not African ancestry [29]. Associations have also been reported for the angiotensin-converting enzyme gene with leisure-time activity [30] and the melanocortin-4 receptor gene with daily activity levels, independent of sex, age, and body mass index (BMI) [31]. A 2021 review by Aasdahl et al. concluded that these genes consistently relate to physical activity or sedentary behavior [32]. As research in this field continues to evolve, it is reasonable to expect that additional genes involved in the regulation of physical activity will be identified, further supporting the idea that multiple biological mechanisms contribute to its regulation. In light of the 24 h activity cycle theory [33], it is also important to consider occupation as a limiting factor for physical activity, one that is not always flexible, when analyzing the reorganization of SED, NEPA, and sleep resulting from the practice of physical exercise. Since a day always consists of 24 h, it is reasonable to assume that adding a physical exercise routine will have varying impacts on the reorganization of daily activities depending on the type of occupation and whether one is employed. Therefore, occupation should be considered among the potential determinants and/or moderators of SED, NEPA, and sleep reorganization. Due to the lack of a comprehensive whole-body approach to this issue, particularly in BCS, we aimed to address this gap by comparing data collected from two different studies that employed comparable materials and methods. This was undertaken despite the well-established importance of aerobic physical exercise in both the general population [34] and BCS [12,13], as it may also negatively affect the reorganization of SED, NEPA, and sleep.

While one of the studies, titled “*Al passo con la tua salute*”, included non-oncologic postmenopausal women, the other, titled “*Allenarsi per la salute*”, involved BCS. Both studies examined the effects of three months of walking (W) or Nordic walking (NW) workouts on participants’ psychophysical health and the reorganization of SED, NEPA, and sleep; however, the study conducted on BCS also incorporated strength and myofascial workouts, which are not addressed in this manuscript. As a consequence, the intervention arms related to these workouts were not included in the analysis.

The aim of the study was to characterize the reorganization of SED, NEPA, and sleep in BCS after three months of aerobic training, compared to non-oncologic postmenopausal women, while considering factors such as age, occupation, weight status, baseline physical activity and sleep levels, workout volume and type, pharmacological treatments, and whether menopause was natural or medically induced.

We hypothesized that, because SED and NEPA are influenced by a range of biological, psychological, cultural, and occupational factors, a three-month program of supervised aerobic exercise would promote favorable adaptations in these behaviors, along with improvements in sleep, in BCS with induced menopause compared to the other groups. This effect is likely mediated by the compensatory plasticity of the underlying determinants, supporting the maintenance of homeostasis.

## 2. Materials and Methods

### 2.1. Study Design

This is a retrospective, non-randomized, parallel, controlled cohort study involving non-oncologic postmenopausal women from the “*Al passo con la tua salute*” study and BCS who were patients at the Integrative Medicine Clinic of both the “G. Bernabeo” Hospital (Ortona, Italy) and the Department of Medicine and Aging Sciences at the “G. d’Annunzio” University of Chieti-Pescara (Italy). All participants attended adapted and supervised W or NW exercise programs. The “*Al passo con la tua salute*” study aimed to investigate the effects of different types of aerobic exercise in non-oncologic postmenopausal women engaged in W or NW workouts during the spring and summer seasons, from April 2009 to October 2015, in the communities of Montesilvano and Pescara (Italy), as well as at the “G. d’Annunzio” University of Chieti-Pescara (Chieti, Italy). After a phone interview to confirm eligibility, selected participants underwent a multidisciplinary evaluation, including a medical examination, assessment of cardiovascular and metabolic health (i.e., blood pressure, resting heart rate, doppler echocardiography, and plasma metabolic analytes), anthropometry and bioelectrical impedance analysis, cardiovascular fitness testing (i.e., graded maximal exercise test), evaluation of daily physical activity and dietary habits, and measurements of SED, NEPA, and sleep through both objective physical activity monitors and subjective nutritional behavior reports. All baseline tests (T_0_) were repeated after three months of training (T_1_) [16,35]. The “*Al passo con la tua salute*” study was approved by the internal review board of the Pescara province health authority (#1070, approved on 24 October 2008), and all participants provided written informed consent.

The “*Allenarsi per la salute*” study, linked to the Integrative Medicine Clinic of both the “G. Bernabeo” Hospital (Ortona, Italy) and the Department of Medicine and Aging Sciences at the “G. d’Annunzio” University of Chieti-Pescara (Italy), provided integrative support for BCS during their follow-up phase. Participants engaged in W or NW workouts during the spring and summer seasons from April 2017 to October 2019, either along the life path of “G. Bernabeo” Hospital or at the “G. d’Annunzio” University. The Integrative Medicine Clinic offers personalized integrative therapies for BCS, including guidance on physical activity, rest, nutrition, body composition, acupuncture, immunological, endocrine, and metabolic assessments (i.e., via blood and/or saliva), psychotherapy, mindfulness, and both indoor and outdoor supervised adapted physical exercise. Following a multidisciplinary T_0_ evaluation, participants wishing to join the adapted and supervised exercise programs were required to obtain medical clearance from an oncologist, physiatrist, and sports medicine specialist. The T_0_ assessment included a comprehensive battery of tests to evaluate lifestyle (i.e., objective recording of SED, NEPA, and sleep; subjective nutritional behavior), body composition (i.e., anthropometry and bioelectrical impedance), cardiovascular and metabolic health (i.e., blood pressure, resting heart rate, heart rate variability, plasma metabolic analytes), adrenal balance (i.e., salivary cortisol and dehydroepiandrosterone sulfate), psychological and quality of life status, via validated questionnaires. Physical fitness tests assessing balance, flexibility, strength, and aerobic capacity were performed after medical approval. If baseline tests were conducted more than one month prior to the start of the workouts, they were repeated to ensure current measurements. All tests were repeated at the end of the training period.

For the purpose of this study, we focused on anamnesis and test results relevant to body mass, stature, and objectively recorded SED, NEPA, sleep, and workout attendance, measures that were collected consistently across both study protocols.

The “*Allenarsi per la salute*” study was approved by the local Ethics Committee (#312/2015, approved on 3 December 2015), and all participants provided written informed consent.

In both studies, the T_1_ objective measurements of SED, NEPA, and sleep were conducted during the two weeks preceding the end of the workout periods, including both training and non-training days. This approach allowed for a more comprehensive characterization of the behavioral responses of the considered variables to the introduction of exercise.

### 2.2. Study Participants

The “*Al passo con la tua salute*” study included 156 postmenopausal women (mean age 58 ± 6 years) with no history of cancer (Meno). Inclusion criteria for participating in the adapted and supervised W or NW sessions were age under 65 years, postmenopausal status, and medical eligibility for W or NW. Exclusion criteria included the use of nutritional supplements or restrictive diets, hormone replacement therapy, endocrine disorders, a history of diabetes mellitus, pulmonary, cardiovascular, or orthopedic conditions limiting the ability to perform W or NW, pharmacological treatments that could directly or indirectly influence the study variables, and participation in any regular exercise program in the two years preceding the study. Women were classified as postmenopausal if they had spontaneous amenorrhea for at least 12 months and plasma estradiol levels below 20 pg/mL.

The study involving patients from the Integrative Medicine Clinic of both the “*G. Bernabeo*” Hospital (Ortona, Italy) and the Department of Medicine and Aging Sciences at the “*G. d’Annunzio*” University of Chieti-Pescara (Italy) included 168 breast cancer survivors (BCS) (mean age 53 ± 9 years). To be eligible for participation in the adapted workout sessions, participants had to meet the following criteria: age between 30 and 70 years, 6 to 48 months post-breast surgery, current hormone therapy, and medical eligibility for W or NW. Exclusion criteria included ongoing chemotherapy or radiation therapy; past or present diagnosis of endocrine disorders; medical conditions limiting the ability to perform W or NW; disorders affecting nutrient absorption, digestion, or assimilation; chronic use of sleep medication at the time of baseline assessment; current pharmacological treatment for depression or anxiety; and participation in any structured exercise program within six months prior to or during the baseline assessment. The term “*current*” refers to the date of each participant’s T_0_ evaluation.

To optimize the length of the manuscript, only the materials and methods relevant to the topic of this paper will be described below.

### 2.3. Interventions

#### 2.3.1. General Characteristics of the Workouts

The Rating of Perceived Exertion (RPE) scale, validated in both healthy populations [36] and clinical conditions [37,38], was used to prescribe and monitor workout intensity. The scale was introduced to participants prior to the start of the training program and reinforced throughout the intervention. Each workout included a warm-up, central, and cool-down phase. The warm-up consisted of a gradual increase in walking speed until the target intensity was reached, while the cool-down involved a progressive decrease in walking pace until RPE fell below 10. Two kinesiologists supervised all sessions, monitored workout intensity using the talk test [39], recorded participant attendance, and tracked any injury occurrence.

Although the RPE scale requires participant familiarization, it was chosen due to its low cost and ability to allow self-regulation of walking intensity. This is particularly useful when aerobic fitness improves over time or following a demanding day, as it enables individuals to adjust their effort accordingly while maintaining target intensity levels.

To minimize seasonal variability in physical activity patterns [40], the intervention was conducted during late spring and summer. Attendance was recorded as a percentage of total sessions, and total workout volume was calculated to reflect changes in both duration and intensity over time. Workout volume was defined as the sum of the products of session duration (in minutes) and session intensity (RPE points) for all attended sessions. Since intensity was prescribed using RPE ranges (e.g., 10–11, 12–13, 13–14), the midpoint value of each range (e.g., 10.5, 12.5, 13.5) was used to compute session volume.

#### 2.3.2. Walking Workouts

A 13-week supervised walking training program was implemented for postmenopausal non-oncologic women, with a frequency of four sessions per week. Each session included three phases: a 5 to 10 min warm-up, a central phase, and a 5 to 10 min cool-down. The duration and intensity of the workouts were progressively adjusted over the course of the program. During the first month, participants performed 40 min sessions at an RPE of 10–11. In the second month, the duration increased to 50 min while maintaining the same intensity. In the final five weeks, the intensity was progressively increased to reach an RPE of 13–14, while the session duration remained unchanged (Table 1).

Breast cancer survivors participated in a 12-week supervised walking training program with a frequency of three sessions per week. Each workout consisted of a 15 min warm-up, a 45 min central phase, and a 10 min cool-down. The intensity of the program was progressively adjusted based on the training month. Specifically, participants trained at an RPE of 10–11 during the first month, 12–13 in the second month, and 13–14 in the third month (Table 1).

#### 2.3.3. Nordic Walking Workouts

Both non-oncologic postmenopausal women and BCS completed 26 full NW sessions, conducted three times per week, in addition to 10 preliminary sessions focused on learning correct NW technique over a three-week period. This technical training followed the guidelines of the International Nordic Walking Federation. Each full NW session followed a consistent structure: a 15 min warm-up, a 45 min central phase, and a 10 min cool-down. For non-oncologic postmenopausal women, the warm-up and cool-down included general exercises recommended by the International Nordic Walking Federation. In the BCS group, the Isa Method was integrated into the warm-up, while both the Isa Method and stretching exercises were included in the cool-down phase [41,42]. The Isa Method included a series of dynamic exercises specifically designed for BCS, serving as preparatory movements for NW and targeting issues such as lymphedema and arthralgia. Training intensity progressed over the course of the 12-week program: participants exercised at an RPE of 10–11 from weeks 1 to 4, 12–13 from weeks 5 to 8, and 13–14 from weeks 9 to 12 (Table 1).

### 2.4. Outcomes

#### 2.4.1. Anthropometry

While participants were in a fasting state, anthropometric measurements were taken by a Level 3 anthropometrist certified by the International Society for the Advancement of Kinanthropometry (ISAK). Body weight and standing height were measured to the nearest 0.1 kg and 0.1 cm, respectively, using a stadiometer with a balance-beam scale (Seca 220, Seca, Hamburg, Germany), with participants barefoot and wearing light clothing [43]. Body mass index (BMI) was computed using the body weight (kg)/stature (m)^2^ formula.

#### 2.4.2. Sedentary Time, Non-Exercise Physical Activity, and Sleep Measurements

Sedentary time, NEPA, and sleep were assessed over three to seven consecutive days in a free-living environment using SenseWear Pro2, Pro3, or Mini armbands (BodyMedia Inc., Pittsburgh, PA, USA), depending on the study period. Each measurement period included at least one full weekend day. T_1_ measurements were carried out on the same days as those at T_0_ and included one to three training days, depending on the study period. Personal information, such as sex, age, height, weight, smoking status, and handedness, was integrated with data collected by the device’s sensors, which include a triaxial accelerometer, skin and near-body temperature sensors, heat flux, and galvanic skin response. The following parameters were extracted: average daily physical activity (expressed in METs); light-intensity physical activity time (LIPAT), time spent in activities with intensity > 1.5 and <3 METs; moderate-intensity physical activity time (MIPAT), time spent in activities with intensity ≥ 3 and ≤6 METs; vigorous-to-very-vigorous physical activity time (V–VIPAT), time spent in activities with intensity > 6 METs. Sedentary time was defined as time spent in activities with intensity ≤ 1.5 METs, excluding sleep and time spent in bed at night. LIPAT, MIPAT, and V–VIPAT, as well as SED, were expressed in daily minutes. Sleep-related outcomes included nighttime in bed, nocturnal sleep duration, and sleep efficiency, calculated as the ratio of sleep duration to time in bed multiplied by 100. Daytime nap periods were categorized as SED [44,45,46,47,48].

The sleeping diary was used to track bedtimes and wake-up hours, as well as identify naps. All day long, except for bathing, participants wore their monitors. If participants wore the multisensory device for at least 960 min per day, their registrations were considered valid [49].

### 2.5. Randomization

In the “*Al passo con la tua salute*” study, non-oncologic participants were not randomized into the W or NW subgroups, as the two exercise modalities were implemented in separate time blocks. Consequently, participants were assigned to either the W or NW group based on the period in which they were recruited.

In the “*Allenarsi per la salute*” study, BCS, the allocation of BCS to either the W or NW subgroup was based on medical eligibility.

### 2.6. Data and Statistical Analyses

#### 2.6.1. Linear Mixed Effects

In order to test the hypothesis of whether adherence to W or NW PA program affected SED and/or NEPA in the three study groups (i.e., Meno; BCS with natural menopause, BCS_Meno; and BCS with medically induced menopause, BCS_Ind_Meno), and if so, by how much, we designed an R script that used Linear Mixed Effects (LME) modeling [50]. However, before choosing LME, the effects of random factors were tested. The final model was assessed graphically and statistically and tested via bootstrapping as described in Bates et al. [50]. Moreover, in order to further control differences at baseline and confounds, ANCOVA-style LMEs were additionally used.

As illustrated in the previous paragraphs, women in this study were tested before and after the PA intervention (T_0_-T_1_ design). Several covariates were considered: age, BMI, hypertension therapy, thyroid therapy, sleep, and METs. Moreover, the two macro categories of hormonal therapy, aromatase inhibitors vs. antiestrogens, and cancer therapy, chemotherapy, and radiotherapy were also accounted for.

Non-Exercise Physical Activity was defined as LIPAT + MIPAT + V-VIPAT. However, T_1_ NEPA had to be corrected by LIPAT, MIPAT, and V-VIPAT measured during the exercise sessions. This correction was performed by accounting for the minutes of exercise in the various phases of each training session. The correction for the BCS natural menopause as well as for the BCS induced menopause groups was –10.71 min from LIPAT and –19.28 min from MIPAT, while V-VIPAT, after verification, was assumed to be zero during the exercise. For the Meno group, the correction varied according to training and monitoring modalities. In W sessions, when monitoring occurred three times per week, the correction was calculated as 25 min for LIPAT and 50 min for MIPAT, divided by three (i.e., one training day was monitored), whereas for five monitoring days per week, the same values were multiplied by three and divided by five. For NW, the same approach was applied, but the central phase was 45 min instead of 50. V-VIPAT was included in post-NEPA, although its contribution was only 1.18% of the total activity.

Distribution for response variable (SED and NEPA) at T_0_ and T_1_ was explored, showing normality. However, the data were rather imbalanced towards the healthy menopause group. Authors are aware that LME models can cope with imbalanced data; however, extra attention was paid to this aspect. We quantified baseline imbalance using standardized mean differences (SMDs). Unadjusted SMDs indicated a large imbalance for age (max SMD = 2.38) and BMI (max SMD = 0.48).

Bivariate summaries were used to explore predicting variables and the possible covariates using the *ggpairs* function [51]. Variables that correlated with the response variables were selected as covariates. If covariates correlated with one another, we selected the dominant. For the final LME models, multicollinearity was assessed using variance inflation factors (VIFs) by means of the *check collinearity* function of the *performance* package.

Missing data analysis was performed using the *naniar* package [52]. This resulted in a proportion of 1% missing data (72 out of 7480). This resulted in a proportion of 1% missing data (72 out of 7480). Of these, 23 data points came from post-test NEPA, 16 data points from the BCS_Meno group, and only 1 data point from post-intervention sedentary assessment, and other variables. Given the low rate of missing data, the authors decided not to impute them. Data were then converted to a long format to analyze the pre- and post-intervention factors. Rescaling is very important in LME models because it can improve model performance and interpretability [53]. Thus, the independent variable adherence and the covariable BMI were centered by subtracting every single value from the variable’s mean value, resulting in a variable with a mean equal to 0 and SD in the original scale of those variables. However, for the covariate sleep and METs, rescaling by the mean value did not solve the issue. Thus, rescaling was achieved by further dividing centered sleep and METs variables by their standard deviation.

An initial random intercepts model was made to assess whether two categorical variables, namely subjects and groups, were in fact random variables. After running the whole analysis with the group variable (three levels) as a random factor, it was decided to remove it, and it will no longer be shown as such. Indeed, Harrison et al. [53] warned about having < 5 groups when a group is used as a random factor. The unconditional models for SED and NEPA as dependent variables and subject as a random factor showed that the total variability of SED attributable to differences among subjects was 44% and of NEPA was 48%. Given the high variability generated by the variable subjects as a random factor, it was justified to keep it as a random factor.

Two Unconditional Growth Models introduced adherence, time (T_0_-T_1_), and group as predictor variables for SED and NEPA (1)

(1) $$\begin{array}{ll} {SED}_{ij}\left[or\;{NEPA}_{ij}\right] & =\beta_{0}+\beta_{1}\cdot {Group}_{i}+\beta_{2}\cdot {Time}_{i}+\beta_{3}\cdot {Adherence}_{i}+\beta_{4}\cdot \left({Group}_{i}\cdot {Time}_{i}\right)\\ & +\beta_{5}\cdot \left({Group}_{i}\cdot {Adherence}_{i}\right)+\beta_{6}\cdot \left({Time}_{i}\cdot {Adherence}_{i}\right)+\beta_{7}\\ & \cdot \left({{Group}_{i}\cdot Time}_{i}\cdot {Adherence}_{i}\right)+b_{0j}+\varepsilon_{ij} \end{array}$$

where $\beta_{0}$ was the overall intercept (fixed effect), $\beta_{1}$ to $\beta_{7}$ were the fixed effect coefficients, $b_{0j}$ was the random intercept for each individual, and $\varepsilon_{ij}$ the residual error term.

Eventually, two final models, including all covariates, were built as follows:

(2) $$\begin{array}{ll} {SED}_{ij}\left[or\;{NEPA}_{ij}\right] & =\beta_{0}+\beta_{1}\cdot {Age}_{i}+\beta_{2}\cdot {Bmi}_{i}+\beta_{3}\cdot {Hormonal\;Therapy}_{i}+\;\beta_{4}\\ & \;\cdot {Radiotheraphy}_{i}+\;\beta_{5}\;\cdot {Chemotheraphy}_{i}+\;\beta_{6}\;\cdot {Treat4Hypertens}_{i}+\beta_{7}\\ & \cdot {Sleep}_{i}+{\beta_{8}\cdot {Mets}_{i}+\beta_{9}\cdot {Group}_{i}+\beta_{10}\cdot {Time}_{i}+\beta_{11}\cdot {Adherence}_{i}+\beta }_{12}\\ & \cdot \left({Group}_{i}\cdot {Time}_{i}\right)+\beta_{13}\cdot \left({Group}_{i}\cdot {Adherence}_{i}\right)+\beta_{14}\\ & \cdot \left({Time}_{i}\cdot {Adherence}_{i}\right)+\;\beta_{15}\cdot \left({{Group}_{i}\cdot Time}_{i}\cdot {Adherence}_{i}\right)+\beta_{16}\\ & \cdot \left({Age}_{i}\cdot {Adherence}_{i}\right)+\beta_{17}\cdot \left({Bmi}_{i}\cdot {Adherence}_{i}\right)\\ & +\beta_{18}\cdot \left({Hormonal\;Therapy}_{i}\cdot {Adherence}_{i}\right)+\beta_{19}\\ & \cdot \left({Radiotheraphy}_{i}\cdot {Adherence}_{i}\right)+\beta_{20}\cdot \left({Chemotheraphy}_{i}\cdot {Adherence}_{i}\right)\\ & +\beta_{21}\cdot \left({Treat4Hypertens}_{i}\cdot {Adherence}_{i}\right)+\beta_{22}\cdot \left({Sleep}_{i}\cdot {Adherence}_{i}\right)+\beta_{23}\\ & \cdot \left({Mets}_{i}\cdot {Adherence}_{i}\right)+b_{0j}+\varepsilon_{ij}a \end{array}$$

where $\beta_{0}$ was the overall intercept (fixed effect), $\beta_{1}$ to $\beta_{23}$ were the fixed effect coefficients, $b_{0j}$ was the random intercept for each individual, and $\varepsilon_{ij}$ the residual error term (extract of R code for LME models can be found in Table S1). The LME model for SED explained 57.4% of the variance at the fixed-effect level and 68.9% when including random effects, and in the case of NEPA, 67.5% of the variance was explained at the fixed-effect level and 75.6% when including random effects. The ICC indicated that 27% of the total variance in SED was attributable to differences between participants and 25% for NEPA.

These two growth models with and without covariates were compared according to the change in the deviance, which is the likelihood ratio test statistic. Maximum likelihood (*l*) is a function of the observations and the model parameters; it returns a measure of the probability of looking at a particular observation, Y, given a set of model parameters β and$\;b_{0j}$, and $\varepsilon_{ij}$ is the unexplained variance. Its output is a $\chi^{2}$ value which represents the difference in deviance between successive models, as well as *p*-values based on likelihood ratio test comparisons. Thus, if significant, it means that adding the parameters to the new model (e.g., Equation (2)) caused a drop in deviance, and this means that the new model explains significantly better by ($\chi^{2}\%$) the Y [50,54]. $\chi^{2}$ for the two ${SED}_{ij}$ and ${NEPA}_{ij}$ models were large (459.8 and 580.7, respectively; both *p* < 0.001), hence models with covariates were superior to models without covariates. Parametric bootstraps were used to better approximate the distribution of the likelihood test statistics and produce more accurate *p*-values by simulating data under the null hypothesis [55]. Finally, diagnostic steps were taken to assess fixed and random effects as described in Bates et al. [50]. Several diagnostic plots were used to assess fixed factors. The scatter plot of fitted values vs. residuals was linear, while the scatter of fitted values vs the squared absolute value of residuals showed homoscedasticity. A *qqmath* plot showed that the empirical distribution was consistent with the theoretical distribution. An autocorrelation showed that data points were not related to preceding or following values. Autocorrelation was less than 0.10 in magnitude [56]. Profile function assessed as the best possible fit. Then we assessed random effects with conditional modes of the random effects. The (one-tailed) posterior predictive *p*-value > 0.05 indicated that the models represented the data adequately. Similarly, LME was used to assess sleep duration *group* × *time* interaction and main effects, accounting for subjects as a random factor. Moreover, because SED, NEPA, and sleep are parts of a whole, a compositional data analysis (CoDA) was implemented using isometric Log-Ratio (ILR) transforms. Then, time-reallocation scenarios (effect of +30 min sleep on SED and NEPA) were assessed. Specifically, we modeled sleep, SED, NEPA, and Other (i.e., when the device was not worn) as a 4-part composition summing to 1,440 min, we applied zero replacement, and computed ILR coordinates using an interpretable sequential binary partition (*compositions* and *coda.base* packages).

#### 2.6.2. Exercise Type, Occupation, and Time from Intervention Effects

Since women in this study underwent W or NW interventions, we assessed whether one or the other type of intervention affected adherence to the PA program, as well as T_1_ NEPA, SED, and sleep. Furthermore, the role of occupation (i.e., housewife, blue-collar, white-collar), and months from the operation on adherence and T_1_ NEPA, SED, and sleep were checked. Because ANOVA’s assumptions were violated, the non-parametric Scheirer–Ray–Hare test was used [57]. This was followed up by the aligned rank transform (ART) model, simple effects were examined using estimated marginal means (EMMs) with pairwise contrasts computed via the *emmeans* package. Holm’s method was applied to adjust *p*-values for multiple comparisons. Regression analysis was used to understand whether the time from intervention correlated with the variable of interest [58].

## 3. Results

### 3.1. Descriptive Statistics

The BCS_Ind_Meno group was significantly younger, had a lower BMI, and a lower number of women treated for hypertension than the other two groups (Table 2). Whereas the Meno group had a shorter sleep duration, a higher number of steps, and METs at baseline than the BCS groups (Table 2). The majority of the BCS_Meno was on aromatase inhibitors, and 38% on antiestrogens. The majority of BCS_Ind_Meno was on antiestrogens and GnRH agonists (Table 3).

### 3.2. Correlations

Adherence and training volume basically had the same meaning, as their correlation was r = 0.992 (*p* < 0.001). Thus, we decided to select adherence as the main predicting variable.

Adherence and training volume did not correlate with baseline SED (r = −0.032 and r = −0.049, respectively) nor with NEPA (r = −0.038 and r = −0.007, respectively). BMI seemed an ideal covariate candidate as it correlated with baseline SED and NEPA and with age (r = 0.377, r = −0.359, *p* < 0.001; and r = 0.120, *p* < 0.05, respectively). Hypertension therapy seemed to affect only the healthy control, the Meno group, which could be an important confound. Sleep did correlate with baseline SED and NEPA (r = −0.133, *p* < 0.05; r = −0.198, *p* < 0.001) and showed a strong correlation with METs (r = −0.152, *p* < 0.001). METs, baseline SED, and NEPA strongly correlated (r = −0.701, r = 0.801, *p* < 0.001).

### 3.3. LME of SED, NEPA, and Sleep

**Figure 1:**
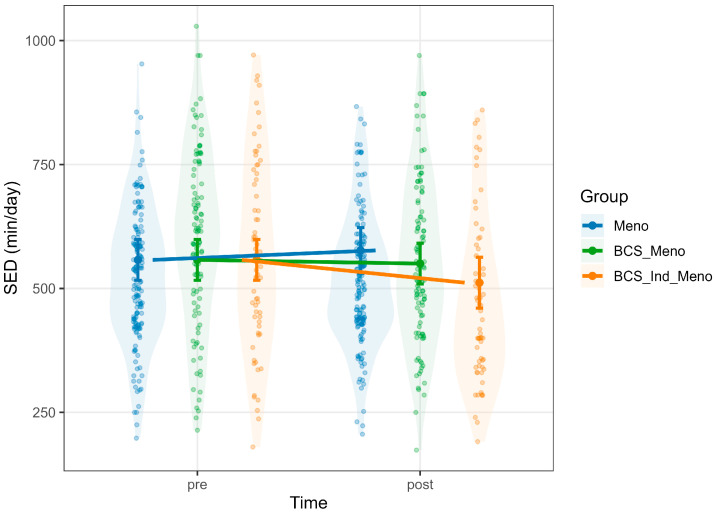
Predicted values of SED. Note: each panel displays violin plots representing the distribution of raw data for each group at each time point. Model-predicted means, derived from mixed-effects models and adjusted for relevant covariates, are shown with vertical error bars indicating 95% confidence intervals. Group-colored lines connect pre- and post-predicted means to highlight within-group changes over time. Meno, blue; BCS_Meno, green; BCS_Ind_Meno, orange.

Levels of SED were reduced by higher METs (Estimate = −115.39, 95% CI [−126.38, −104.40], *p* < 0.001) and sleep duration (Estimate = −43.71, 95% CI [−52.64, −34.78], *p* < 0.001). Furthermore, there was a significant interaction between *group* × *time* for BCS_Ind_Meno (Estimate = −65.11, 95% CI [−102.82, −27.40], *p* = 0.001; Table S2), indicating that the BCS_Ind_Meno group had a significant decrease in SED after the intervention (Figure 1).

**Figure 2:**
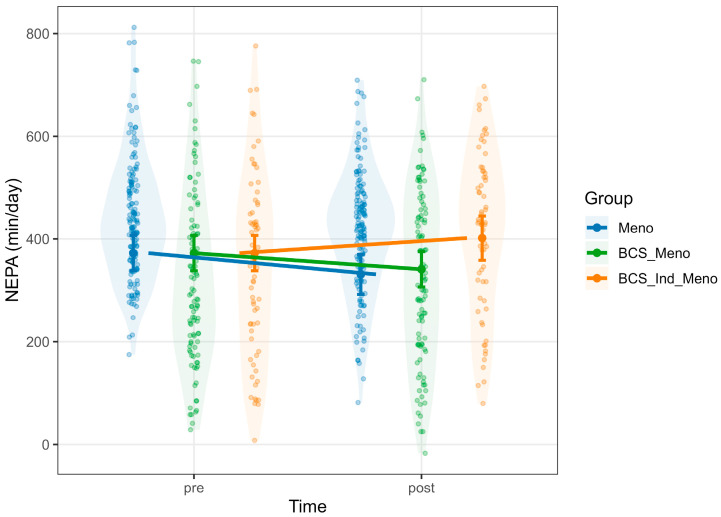
Predicted values of NEPA. Note: each panel displays violin plots representing the distribution of raw data for each group at each time point. Model-predicted means, derived from mixed-effects models and adjusted for relevant covariates, are shown with vertical error bars indicating 95% confidence intervals. Group-colored lines connect pre- and post-predicted means to highlight within-group changes over time. Meno, blue; BCS_Meno, green; BCS_Ind_Meno, orange.

**Figure 3:**
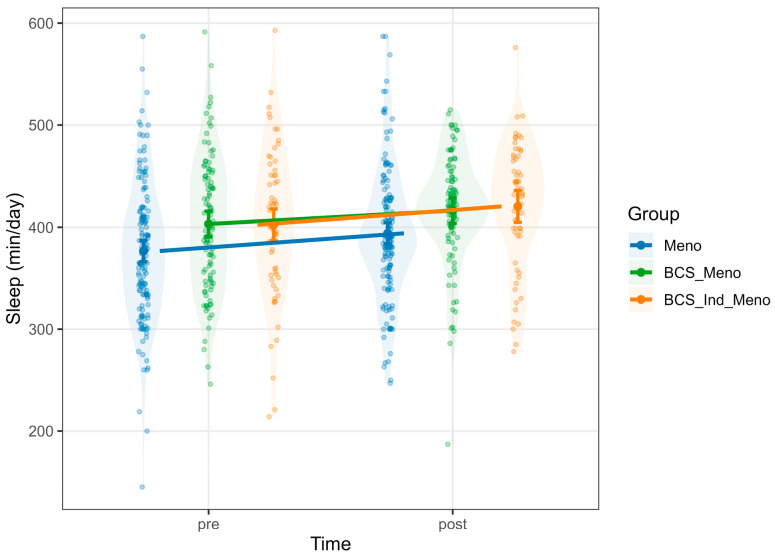
Predicted values of sleep. Note: each panel displays violin plots representing the distribution of raw data for each group at each time point. Model-predicted means, derived from mixed-effects models and adjusted for relevant covariates, are shown with vertical error bars indicating 95% confidence intervals. Group-colored lines connect pre- and post-predicted means to highlight within-group changes over time. Meno, blue; BCS_Meno, green; BCS_Ind_Meno, orange.

NEPA was affected positively by METs (Estimate = 121.06, 95% CI [111.81, 130.32], *p* < 0.001), while sleep had a small negative effect on NEPA (Estimate = −8.67, 95% CI [−16.20, −1.14], *p* = 0.017) (Table S3). There was a significant *group* × *time* interaction, where the BCS_Ind_Meno group showed a significant increase in NEPA after the intervention (Estimate = 70.56, 95% CI [38.44, 102.68], *p* < 0.001; Figure 2). A significant main effect of time was found for sleep, indicating a general increase in sleep duration after the intervention (Estimate = 17.39, 95% CI [4.79, 29.98], *p* = 0.007). There was also a main effect of group showing that both BCS_Meno (Estimate = 26.52, 95% CI [10.05, 42.98], *p* = 0.002) and BCS_Ind_Meno (Estimate = 25.66, 95% CI [6.73, 44.60], *p* = 0.008) reported significantly longer sleep compared to the Meno group (Table S4 and Figure 3).

An ANCOVA-style LME, which modeled post-intervention SED while adjusting for baseline SED and covariates (age, BMI, therapies, sleep, METs), yielded concordant effects. Relative to Meno, the BCS_Ind_Meno group showed a significantly larger reduction from baseline (Estimate = −73.4 [95% CI −110.1, −36.7], *p* < 0.001), whereas the BCS_meno group showed a non-significant trend (Estimate = −30.0 [−62.6, 2.6], *p* = 0.071). Adjusted T_1_ differences and estimated marginal means are reported in Table S5. The ANCOVA coefficient for baseline (β = 0.47, *p* < 0.001) indicated appropriate baseline adjustment. Sleep (β = −31.9 per unit, *p* < 0.001) and METs (β = −77.5 per unit, *p* < 0.001) were independently associated with lower SED levels. Consistently, the ANCOVA-style LME for NEPA indicated that baseline NEPA was a strong predictor of post-intervention NEPA (β = 0.39, 95% CI [0.34, 0.45], *p* < 0.001), confirming appropriate adjustment. It showed a significant improvement for BCS_Ind_Meno (Estimate = 77.40 min/day (95% CI [44.99, 109.80], *p* < 0.001). BCS_Meno remained non-significant in both models. METs were a strong positive predictor in both approaches (85.88 [76.84, 94.91], *p* < 0.001), while the negative effect of sleep observed in the standard LME was attenuated in ANCOVA. A higher BMI also showed a small positive effect (β = 2.65, 95% CI [1.07, 4.22], *p* = 0.001; Table S6). Although baseline sleep was rather similar across groups, baseline sleep strongly predicted follow-up (β ≈ 0.63, *p* < 0.001). After adjusting for baseline, model R^2^ increased from 0.05 to 0.47, without altering the main conclusion that *group* × *time* interactions were non-significant (Table S7). Sleep duration increased on average by about 17 min from T_0_ to T_1_ (*p* = 0.002), with no significant *group* × *time* interactions *p* > 0.63, suggesting an overall improvement over time (Table S7).

Furthermore, since three types of armbands were used in these data collections, a model including device type as a covariate was tested. However, adding device type and its interaction with adherence did not significantly improve model fit for either SED (χ^2^(4) = 4.45, *p* = 0.35; AIC: 7704.4 vs. 7707.9; BIC: 7851.2 vs. 7872.5) or NEPA (χ^2^(4) = 2.43, *p* = 0.66; AIC: 7503.5 vs. 7509.0; BIC: 7650.3 vs. 7673.6), indicating that device type did not covary with these outcomes beyond the other covariates considered. Residual collinearity results are shown in Table S8. Importantly, the continuous covariates (i.e., age, BMI, sleep, METs) had low VIFs (≈1–2.5), while moderate VIFs (≈5–10) were associated with time and its interactions, which we expected in a prespecified *group* × *time* × *adherence* structure. The very high raw VIFs occurred for binary treatment indicators (i.e., hormonal therapy, radiotherapy) and their interactions. This was due to dummy coding rather than redundancy among continuous covariates.

### 3.4. CoDA, ILR, and Time Reallocation

After transforming SED, NEPA, and sleep minutes into compositions and applying log-ratio transformations, and after including the minutes when the device was not worn as *other* category, a time reallocation of +30 min to sleep was used to examine its impact on SED and NEPA. When 30 min were proportionally reallocated from SED and NEPA, both decreased (SED −18.1 min, NEPA −11.9 min). Distributing the 30 min increase across all waking behaviors (SED, NEPA, *other*) led to SED −16.3 min, NEPA −10.7 min, and *other* −3.1 min. Thus, under proportional reallocations, increasing sleep by 30 min primarily displaced SED more than NEPA, reflecting the larger baseline share of sedentary behavior (Figure S1).

### 3.5. Exercise Type, Occupation, and Time from Intervention

Allocation of W and NW amongst the three groups was rather balanced. However, Shapiro–Wilk revealed no normal distributions. When comparing adherence across menopausal groups and training condition (W vs. NW), we found no significant interaction, H(2) = 0.63, *p* = 0.731; partial η^2^ = 0.002, 95% CI 0.000–0.027. There was a significant interaction between groups and W or NW for SED at T1, H(2) = 9.82, *p* = 0.007; partial η^2^ = 0.032, 95% CI 0.009–0.080, indicating that the effect of menopausal status differed by training condition. SED was significantly lower in W compared to NW for the Meno group (estimate = −42.5 min, padj = 0.005), and significantly higher for BCS_Meno (estimate = 43.9 min, padj = 0.036). No significant difference was observed for BCS_Ind_Meno (padj = 0.795). Specifically, in the W group, median SED was 459 min (IQR 284) for BCS_Ind_Meno, 585 min (IQR 259) for BCS_Meno, and 494 min (IQR 182) for Meno. In the NW group, medians were 446 min (IQR 145), 481 min (IQR 133), and 515 min (IQR 146), respectively. Estimated marginal means (EMMs) confirmed these patterns: for Meno, EMM = 144 min W vs. 187 min NW; for BCS_Meno, EMM = 168 vs. 124; for BCS_Ind_Meno, EMM = 158 vs. 152. When accounting for SED at T_0_ and other confounds, as we have done in the ANCOVA-style LME, no influence of training group emerged (Table S5). There were no interactions for T_1_ NEPA, H(2) = 3.74, *p* = 0.154; partial η^2^ = 0.013, 95% CI 0.002–0.053, nor for T_1_ sleep duration, H(2) = 1.15, *p* = 0.563; partial η^2^ = 0.004, 95% CI 0.000–0.034, as confirmed by ANCOVA-style LME (Table S6). 

There was a significant interaction between group and job types (housewife, blue-collar, white-collar), H(4) = 10.60, *p* = 0.031; partial η^2^ = 0.036, 95% CI 0.015–0.092. Follow-up tests revealed that housewives in both the BCS_Ind_Meno (median = 88) and BCS_Meno (median = 88) groups seemed to be more adherent than those in the Meno (median = 71) group, who had similar adherence (padj = 1.00 for all pairs). However, the BCS_Meno group was much smaller, so a possible regression to the mean may be expected. In blue-collar workers (small cell sizes: n = 5 for each BCS group), BCS_Meno showed a significantly higher adherence (median = 92) than Meno, which was the lowest (54) (padj = 0.025). In white-collar workers, differences were modest. Yet BCS_Ind_Meno (median = 85) showed higher adherence than Meno (median = 76) (padj = 0.032). While for post- SED, NEPA and sleep there were no interactions (H(4) = 3.82, *p* = 0.430, H(4) = 2.55, *p* = 0.635, and H(4) = 4.53, *p* = 0.338, respectively). Corresponding rank-based interaction effect sizes were small: partial η^2^ = 0.0126, 0.0088, and 0.0153, respectively.

Finally, there was no correlation between months from the operation and adherence R^2^ = 0.016, *p* = 0.085, post-intervention SED R^2^ = 0.001, *p* = 0.659, post-intervention NEPA R^2^ = 0.001, *p* = 0.169, and post-intervention sleep R^2^ = 0.012, *p* = 0.137.

## 4. Discussion

The first important finding of our study is the confirmation of the positive effect of a 3-month outdoor aerobic exercise program on sleep duration in both BCS and postmenopausal women without cancer [14,15], independent of other variables, with an increase of approximately 17 min. Integrating our results with existing literature, it is reasonable to suggest that BCS who engage in aerobic exercise benefit from the general multifaceted interplay of biochemical, thermoregulatory, and circadian mechanisms, commonly observed in the general population, while also counteracting specific adverse effects related to cancer treatments. In general, aerobic exercise stimulates muscle contractions that trigger the release of peripheral factors, which interact with central nervous system pathways to enhance slow-wave sleep. Concurrently, exercise-induced increases in brain adenosine levels, thermoregulatory adjustments, parasympathetic activation, and improved circadian synchronization act synergistically to promote sleep onset, stability, and depth [59]. In the case of BCS, additional mechanisms may be at play: indeed, literature suggests that increased oxygen consumption during aerobic activity may attenuate pain sensitization by activating endogenous pain inhibitory pathways, ultimately improving sleep quality. This improvement in sleep may, in turn, contribute to enhanced quality of life and reduced cancer-related fatigue, creating a positive feedback loop [60]. The presence of a baseline gap in sleep duration between BCS and postmenopausal women without cancer, where the latter exhibited shorter sleep from the beginning till the end of the study, may be attributed to individual variability in daily sleep requirements for recovery and/or to cancer-related fatigue, which may lead BCS to require more sleep to achieve adequate physiological restoration.

The second important finding of our study is the concurrent change observed in SED, NEPA, and sleep duration in the studied populations following a 3-month outdoor aerobic exercise program. In general, an increase in sleep duration was associated with a reduction in both SED and NEPA, with the decrease in SED being more pronounced than that in NEPA. This finding can be interpreted in light of the 24 h activity cycle theory, which posits that an increase in sleep necessarily reduces the time available for other activities, such as SED and NEPA. When interpreting the NEPA results, it is important to consider that, during the T_1_ measurements, participants also engaged in the scheduled exercise sessions, excluded from NEPA calculation, which were held at fixed times and had to be coordinated with participants’ occupational status and other daily commitments. Taking all this into account, the synchronized pattern of change across these variables can be interpreted as a positive outcome, even though the most favorable result was observed in the BCS_Ind_Meno subsample. Indeed, the BCS_Ind_Meno subsample was the only group to show a simultaneous reduction in SED and an increase in both NEPA and sleep. This result suggests that the BCS_Ind_Meno subsample was a more positive responder compared to the others, decreasing their SED by approximately 65 min/day (effect size = 3.38, large) and increasing their NEPA by about 70 min/day (effect size = 4.16, large). This finding aligns with previous research; for instance, Anderson et al. [61] found that with a 5-year increase in diagnosis age, women were 11–16% less likely to report a positive change in health behaviors. Similarly, Thomas et al. [62] observed that physical activity is often used by young cancer survivors as a coping strategy to restore a sense of normalcy, regain physical fitness, engage in social interactions, and manage or prevent the negative side effects of treatment. This suggests that the BCS_Ind_Meno subsample, in addition to attending exercise sessions, exerted greater active control over the reallocation of physical activity, likely as a consequence of higher intrinsic motivation, mainly related to age and the perceived importance of investing in one’s future, by intentionally reducing SED and increasing NEPA. The clear implication of this finding is that optimizing the intervention requires personalization, not only in selecting the most appropriate type of physical exercise and designing its proper periodization [63], but also in adopting psychological strategies tailored to the individual’s age and condition. In this context, a one-size-fits-all approach is inadequate.

The third, fourth, and fifth main findings of our study hold a more practical importance. The third finding relates to the principle that, although starting physical exercise as early as possible is the primary goal, it is never too late to intervene. In fact, according to our results, there were no significant correlations between the time elapsed since surgery and the date of enrollment in the study with respect to METs, SED, NEPA, and sleep, nor regarding changes in these variables by the end of the study or exercise attendance. The fourth finding highlights the importance of considering a patient’s occupation when prescribing exercise, as it can represent a barrier to participation, particularly evident among our blue-collar participants. This is especially relevant for centers offering supervised exercise programs, where the goal is to safely introduce patients to physical exercise practice. In most cases, occupation is a non-modifiable factor that influences exercise attendance, alongside other key parameters involved in exercise prescription and periodization [63]. To account for the observed differences in exercise attendance and to avoid the presence of a confounding factor in our results, all analyses were conducted while controlling for the effect of exercise attendance. The fifth finding highlights the different effects of W and NW, despite similar levels of exercise attendance. According to our results, the studied disciplines, together with the pharmacological treatments per se, did not elicit different effects on SED, NEPA, or sleep reallocation. In our opinion, the significant result of the non-parametric Scheirer–Ray–Hare test for SED was mainly due to its failure to account for the T_0_ values. Indeed, when the ANCOVA-style LME was performed, the observed significance disappeared.

This study provides important insights but also highlights the need for further research in the field of optimizing physical activity counseling for psychophysical health. Building on the evidence related to the optimization of LIPAT and MIPAT in the absence of physical exercise [64], this work provides important practical guidelines to apply when aerobic exercise is included in BCS, in order to optimize its positive effects.

However, this study is not without limitations. As a retrospective study, data were collected at different points in time, likely contributing to greater variance among the observed variables. In addition, differences in inclusion criteria and total training volumes, resulting in data from studies with different objectives, including the use of devices from the same manufacturer but with progressively improved technology, must be acknowledged as limitations of this study, despite analyses accounting for age, occupation, weight status, device type, baseline physical activity and sleep levels, workout volume and type, pharmacological treatments, and whether menopause was natural or medically induced. For instance, while the “*Al passo con la tua salute*” study was designed to analyze the effects of W and NW, taking into account that NW results in approximately 20–30% higher oxygen consumption than W at the same speed, the “*Allenarsi per la salute*” study focused on ways to safely optimize training time in BCS. Other issues were the imbalance in the dataset and the lack of data regarding changes in nutritional behavior in both groups (Material and Methods S1), which could further affect metabolic compensation and sleep. The authors attempted to mitigate this by employing LMEs. However, certain variables, such as job type, may still be affected by regression to the mean. Additionally, the presence of two types of interventions (i.e., W and NW), although fairly allocated, did not guarantee normality. Nevertheless, the benefits observed in the BCS_Ind_Meno group, as evaluated through LME while accounting for multiple covariates, appear to be quite robust. Future studies should address this limitation using methods such as sensitivity analyses or propensity score adjustment to balance observed covariates between groups.

## 5. Conclusions

The results presented provide an important contribution to the scientific literature on the effects of aerobic physical exercise on SED, NEPA, and sleep in women treated for breast cancer, both premenopausal and postmenopausal, as well as in postmenopausal women without a history of oncological disease. Obviously, these findings are specific to the spring and summer period and should not be generalized to the entire year. However, the significance of these findings goes beyond this. They also offer useful insights for optimizing exercise prescription and programming. Specifically, the integration of supervised outdoor aerobic exercise, performed in either W or NW modality over a three-month period, into women’s weekly routines appears to increase sleep duration, regardless of clinical condition. In contrast, the optimal reallocation of SED, NEPA, and sleep, characterized by a reduction in SED and an increase in the other two variables, was observed only in the BCS_Ind_Meno subgroup. This finding reinforces the importance of proper exercise prescription and programming, which must always be grounded in a thorough understanding of the individual. This includes not only medical history, but also personal barriers and motivations related to exercise, as well as motor preferences, especially in postmenopausal women, and even more so in blue-collar individuals, who typically show lower intrinsic motivation and face greater obstacles to regular physical activity.

## Figures and Tables

**Table 1:** Description of the interventions.

Characteristics	Meno W	Meno NW	BCS W	BCS NW
Month 1				
Training sessions per week	4	3	3	3
Warm-up	5–10 min	15 min	15 min	15 min
Central phase	40 min at RPE of 10–11	45 min at RPE of 10-11	45 min at RPE of 10–11	45 min at RPE of 10–11
Cool-down	5–10 min	10 min	10 min	10 min
Month 2				
Training sessions per week	4	3	3	3
Warm-up	5–10 min	15 min	15 min	15 min
Central phase	50 min at RPE of 10–11	45 min at RPE of 12–13	45 min at RPE of 12–13	45 min at RPE of 12–13
Cool-down	5–10 min	10 min	10 min	10 min
Month 3				
Training sessions per week	4	3	3	3
Warm-up	5–10 min	15 min	15 min	15 min
Central phase	50 min at RPE of 13–14	45 min at RPE of 13–14	45 min at RPE of 13–14	45 min at RPE of 13–14
Cool-down	5–10 min	10 min	10 min	10 min
Month 4				
Training sessions per week	4 (just for 1 week)	-	-	-
Warm-up	5–10 min	-	-	-
Central phase	50 min at RPE of 13–14	-	-	-
Cool-down	5–10 min	-	-	-

Note: Meno, postmenopausal women with no history of cancer; BCS, breast cancer survivors; W, walking; NW, Nordic walking.

**Table 2:** Descriptive characteristics of the participants.

Characteristic	N	Overall N = 324 ^1^	Meno n = 156 ^1^	BCS_Meno n = 102 ^1^	BCS_Ind_Meno n = 66 ^1^	*p*-Value ^2^
Age (Years)	324	56 (50, 61)	58 (54, 63)	58 (53, 63)	46 (43, 48)	<0.001
BMI (kg/m^2^)	324	26.0 (23.5, 29.4)	26.0 (24.2, 28.6)	27.1 (24.2, 30.7)	24.4 (21.6, 28.0)	0.002
Antihypertensive therapy	324					<0.001
No		196 (60%)	84 (54%)	57 (56%)	55 (83%)	
Yes		128 (40%)	72 (46%)	45 (44%)	11 (17%)	
Therapy for hyper or hypothyroidism	324					0.12
No		301 (93%)	143 (92%)	93 (91%)	65 (98%)	
Yes		23 (7.1%)	13 (8.3%)	9 (8.8%)	1 (1.5%)	
Sleep (minutes)	323	390 (342, 440)	376 (334, 416)	399 (350, 450)	402 (354, 451)	0.002
Steps (#)	323	9989 (7271, 12,685)	11,074 (9003, 13,868)	8707 (6848, 11,401)	8435 (6838, 11,399)	<0.001
METs (#)	323	1.36 (1.20, 1.50)	1.40 (1.30, 1.60)	1.27 (1.16, 1.40)	1.33 (1.20, 1.50)	<0.001

Note: Meno, postmenopausal women with no history of cancer; BCS_Meno, BCS with natural menopause; BCS_Ind_Meno, BCS with medically induced menopause; BMI, body mass index; METs, Metabolic Equivalent of Task; #, number. ^1^ Median (Q1, Q3); n (%). ^2^ Kruskal–Wallis rank sum test; Pearson’s Chi-squared test; Fisher’s exact test.

**Table 3:** Oncological therapies of participants.

Characteristic	N	Overall N = 316 ^1^	Meno n = 148 ^1^	BCS_Meno n = 102 ^1^	BCS_Ind_Meno n = 66 ^1^	*p*-Value ^2^
**Chemotherapy**	316					<0.001
No		132 (42%)	0 (0%)	90 (88%)	42 (64%)	
Yes		36 (11%)	0 (0%)	12 (12%)	24 (36%)	
Non-oncologic women		148 (47%)	148 (100%)	0 (0%)	0 (0%)	
**Radiotherapy**	316					<0.001
No		38 (12%)	0 (0%)	24 (24%)	14 (21%)	
Yes		130 (41%)	0 (0%)	78 (76%)	52 (79%)	
Non-oncologic women		148 (47%)	148 (100%)	0 (0%)	0 (0%)	
**Hormonal therapy**	316					
ARO_inhib		62 (20%)	0 (0%)	62 (61%)	0 (0%)	
ARO_inhib_AnalogGnRH		5 (1.6%)	0 (0%)	0 (0%)	5 (7.6%)	
Antiestrog_AnalogGnRH		61 (19%)	0 (0%)	0 (0%)	61 (92%)	
Antiestrogen		39 (12%)	0 (0%)	39 (38%)	0 (0%)	
Non-oncologic women		149 (47%)	148 (100%)	1 (1.0%)	0 (0%)	

Note: Meno, postmenopausal women with no history of cancer; BCS_Meno, BCS with natural menopause; BCS_Ind_Meno, BCS with medically induced menopause; ARO_inhib, aromatase inhibitors; ARO_inhib_AnalogGnRH, aromatase inhibitors + GnRH analogue; Antiestrog_AnalogGnRH, antiestrogen + GnRH analogue. ^1^ n (%). ^2^ Pearson’s Chi-squared test.

## Data Availability

Data from the present study are available from the corresponding author upon reasonable request.

## Supplementary Materials

The following supporting information can be downloaded at https://www.mdpi.com/article/10.3390/healthcare13192471/s1, Material and Methods S1: Dietary habits recording; Table S1: R syntax for main LME models; Table S2: Linear mixed effect model for SED; Table S3: Linear mixed effect model for NEPA; Table S4: Linear mixed effect model for sleep; Table S5: ANCOVA for SED; Table S6: ANCOVA for NEPA; Table S7: ANCOVA for sleep; Table S8: Multicollinearity diagnostics, Variance Inflation Factors (VIFs) for LME SED and NEPA models; Figure S1: Wake-time composition of NEPA, SED, and OTHER behaviors represented in a ternary plot. References [65,66,67] are cited in the supplementary materials.

## Abbreviations

The following abbreviations are used in this manuscript:

Antiestrog_AnalogGnRH	Antiestrogen + GnRH analogue
ARO_inhib	Aromatase inhibitors
ARO_inhib_AnalogGnRH	Aromatase inhibitors + GnRH analogue
BCS	Breast Cancer Survivors
BCS_Ind_Meno	BCS with medically induced menopause
BCS_Meno	BCS with natural menopause
BMI	Body Mass Index
LIPAT	Light-Intensity Physical Activity Time
LME	Linear Mixed Effects
Meno	Postmenopausal women with no history of cancer
METs	Metabolic Equivalent of Task
MIPAT	Moderate-Intensity Physical Activity Time
NEPA	Non-Exercise Physical Activity
NW	Nordic Walking
RPE	Rating of Perceived Exertion
SED	Sedentary time
V-VIPAT	Vigorous-to-very Vigorous Physical Activity Time
W	Walking
