# Supplementary material for: Aerobic Training-Induced Changes in Sedentary Time, Non-Exercise Physical Activity, and Sleep Among Breast Cancer Survivors and Postmenopausal Women Without Cancer
Source: Healthcare (Basel). 2025 Sep 29;13(19):2471. doi: 10.3390/healthcare13192471 (PMC12524753; doi:10.3390/healthcare13192471)
Supplement: Supplementary file 1 [file healthcare-13-02471-s001.zip › Materials and methods S1.pdf]

Supplementary Materials and Methods

Dietary habits recording

In the *"Al passo con la tua salute"* study, dietary habits were assessed at both T_0 and T_1, without implementing any dietary intervention. A registered dietitian used the WinFood 2.7 software (Medimatica, Colonnella, Italy) to analyze three-day dietary records, covering two weekdays and one weekend day, to estimate caloric intake and dietary patterns. The analysis provided both quantitative data (e.g., kilocalories and grams) and qualitative data (e.g., macronutrient composition of daily meals). Participants recorded the quantities of food consumed using an open-ended form that included instructions for completing the dietary record. Portion sizes were self-measured using kitchen scales or standard household units (e.g., cups and tablespoons). After the recording period, the dietitian reviewed the records with each participant to ensure clarity, identify any missing meals, and gather additional details about food preparation methods.

In the *"Allenarsi per la salute"* study, baseline dietary assessment was combined with a structured dietary intervention. Nutritionists provided individualized Mediterranean-based nutritional counseling aimed at managing glycemia and insulinemia, reducing renal workload, supporting liver and thyroid function, promoting calcium turnover, controlling plasma cholesterol, improving sleep quality, and alleviating hot flashes and arthralgia. Based on individual characteristics identified through anamnesis and clinical evaluations, the nutritional intervention emphasized low-glycemic index, low-energy-density meals rich in vegetables and appropriate protein intake. Educational efforts focused on nutrient quality, proper food pairing and timing, optimal use of olive oil, healthy cooking methods, and correct portion sizing. If needed, a focus on body fat reduction, maintaining the muscle mass, is pursued (1–3). Following each consultation, participants received an individualized written nutritional plan. Additionally, the nutritionist encouraged participants to handwrite the key principles discussed during the visit in order to reinforce recall and adherence. Monthly follow-up visits were scheduled with the same nutritionist, during which the core concepts of the participant's personalized

nutritional approach were reviewed, and a revised nutritional plan was provided based on the participant's feedback and evolving needs.

Throughout the month, participants had the opportunity to contact the nutritionist by phone for clarification, support, or to report the onset of new side effects or health concerns.

References

1. Schwingshackl L, Schwedhelm C, Galbete C, Hoffmann G. Adherence to Mediterranean Diet and Risk of Cancer: An Updated Systematic Review and Meta-Analysis. Nutrients. 2017 Sept 26;9(10):1063.

2. Inglis JE, Lin PJ, Kerns SL, Kleckner IR, Kleckner AS, Castillo DA, et al. Nutritional Interventions for Treating Cancer-Related Fatigue: A Qualitative Review. Nutrition and Cancer. 2019 Jan 2;71(1):21–40.

3. Markellos C, Ourailidou ME, Gavriatopoulou M, Halvatsiotis P, Sergentanis TN, Psaltopoulou T. Olive oil intake and cancer risk: A systematic review and meta-analysis. Caruso C, editor. PLoS ONE. 2022 Jan 11;17(1):e0261649.
