# Supplementary material for: Aerobic Training-Induced Changes in Sedentary Time, Non-Exercise Physical Activity, and Sleep Among Breast Cancer Survivors and Postmenopausal Women Without Cancer
Source: Healthcare (Basel). 2025 Sep 29;13(19):2471. doi: 10.3390/healthcare13192471 (PMC12524753; doi:10.3390/healthcare13192471)
Supplement: Supplementary file 1 [file healthcare-13-02471-s001.zip › Supplementary Figure and Tables.pdf]

Supplementary Table S1. R syntax for main LME models.

Yij	*lmer*
SED	sedentary ~ Group * time * adherence_c + age_c + bmi_c + sleep_z + mets_z + hormonal_therapy + radiotherapy + chemotherapy + tr_hypertension + (1 | id)
NEPA	nepa ~ Group * time * adherence_c + age_c + bmi_c + sleep_z + mets_z + hormonal_therapy + radiotherapy + chemotherapy + tr_hypertension + (1 | id)
Sleep	sleep ~ Group * time + (1 | id)

Notes: Adherence and BMI were mean-centered by subtracting the sample mean and keeping original units. Sleep and METs were standardized (z-scores) by subtracting the mean and dividing by the standard deviation. Models were estimated using the *lme4* package (version 1.1-35.5) in R (version 4.4.0).

Supplementary Table S2. Linear mixed-effect model for SED.

	SED			
Predictors	*Estimates*	*CI*	*p*	*df*
(Intercept)	557.67	514.97 – 600.37	**<0.001**	415.52
cage	0.81	-0.95 – 2.57	0.364	344.42
cbmi	-0.93	-3.40 – 1.53	0.457	395.87
hormonal therapy [2]	22.62	-65.66 – 110.91	0.615	384.99
hormonal therapy [3]	4.92	-42.03 – 51.87	0.837	494.38
hormonal therapy [4]	-21.00	-61.06 – 19.05	0.303	345.77
hormonal therapy [no]	-4.30	-177.07 – 168.47	0.961	340.47
radiotherapy [2]	12.56	-22.45 – 47.58	0.481	340.46

radiotherapy [no]	-19.10	-194.65 – 156.45	0.831	348.24
chemotherapy [2]	-10.82	-47.49 – 25.85	0.562	341.85
hypertension therapy [1]	-16.81	-39.53 – 5.90	0.146	346.04
csleep	-43.71	-52.64 – -34.78	**<0.001**	662.31
cmets	-115.39	-126.38 – -104.40	**<0.001**	663.12
time [post]	19.12	-2.21 – 40.45	0.079	329.43
cadherence	0.15	-0.74 – 1.04	0.738	554.85
group [BCS_Meno] × time [post]	-26.58	-60.08 – 6.92	0.120	324.30
group [BCS_Ind_Meno] × time [post]	-65.11	-102.82 – -27.40	**0.001**	324.69
group [BCS_Meno] × cadherence	-1.51	-4.01 – 0.99	0.234	420.32
group [BCS_Ind_Meno] × cadherence	0.22	-2.05 – 2.48	0.850	446.51
time [post] × cadherence	-0.32	-1.21 – 0.57	0.475	344.91
cage × cadherence	0.06	-0.02 – 0.14	0.117	349.93
cbmi × cadherence	-0.03	-0.17 – 0.10	0.616	381.25
hormonal therapy [2] × cadherence	1.21	-1.29 – 3.71	0.343	358.85
hormonal therapy [4] × cadherence	1.46	-0.69 – 3.60	0.183	342.05
radiotherapy [2] × cadherence	-0.51	-2.37 – 1.34	0.586	341.06
chemotherapy [2] × cadherence	1.32	-0.62 – 3.26	0.181	341.22
tr hypertension [1] × cadherence	0.72	-0.35 – 1.79	0.186	341.08
csleep × cadherence	0.27	-0.11 – 0.66	0.162	661.76
cmets × cadherence	-0.34	-0.84 – 0.16	0.185	661.53
(group [BCS_Meno] × time [post]) × cadherence	0.15	-1.44 – 1.73	0.856	329.44

(group [BCS_Ind_Meno] × time [post]) × cadherence	0.63	-0.98 – 2.24	0.440	327.50
**Random Effects**				
σ^2	7864.84			
$\tau_{00\ id}$	2924.25			
ICC	0.27			
N id	316			
Observations	632			
Marginal R^2 / Conditional R^2	0.574 / 0.689			

Notes: SED, sedentary behavior; cage, centered age; cbmi, centered BMI; hormonal therapy [2], ARO_inhib_AnalogGnRH; hormonal therapy [3], Antiestrog_AnalogGnRH; hormonal therapy [4], Antiestrogen; hormonal therapy [no], Non-oncologic women; radiotherapy [2], Breast Cancer survival who had radiotherapy; radiotherapy [no], Non-oncologic women; chemotherapy [2], Breast Cancer survival who had chemotherapy; hypertension therapy [1], taking drugs for hypertension; csleep, centered sleep; cmets, centered Metabolic equivalent of task; cadherence, centered adherence; BCS_Meno, Breast Cancer survival with natural menopause; BCS_Ind_Meno, Breast Cancer survival with medically induced menopause; σ2, residual variance; τ00 id, random intercept for subjects; ICC, interclass correlation coefficient; N id, number of subjects.

Supplementary Table S3. Linear mixed-effect model for NEPA.

	**NEPA**			
Predictors	*Estimates*	*CI*	*p*	*df*
(Intercept)	372.42	336.78 – 408.07	**<0.001**	418.69
cage	-0.05	-1.51 – 1.42	0.951	344.40
cbmi	1.09	-0.97 – 3.15	0.298	395.51
hormonal therapy [2]	-35.02	-108.65 – 38.62	0.350	386.76
hormonal therapy [3]	-8.99	-48.27 – 30.29	0.653	500.50
hormonal therapy [4]	16.67	-16.70 – 50.04	0.327	345.72
hormonal therapy [no]	-46.44	-190.35 – 97.47	0.526	340.45
radiotherapy [2]	-1.27	-30.43 – 27.90	0.932	340.44
radiotherapy [no]	90.08	-56.19 – 236.35	0.227	348.51
chemotherapy [2]	0.31	-30.23 – 30.86	0.984	341.84
hypertension therapy [1]	2.13	-16.80 – 21.05	0.825	345.97

csleep	-8.67	-16.20 – -1.14	**0.024**	663.88
cmets	121.06	111.81 – 130.32	**<0.001**	661.25
time [post]	-41.29	-59.46 – -23.13	**<0.001**	329.47
cadherence	-0.59	-1.34 – 0.15	0.118	561.03
group [BCS _Meno] × time [post]	9.87	-18.67 – 38.41	0.497	324.31
group [BCS _Ind_Meno] × time [post]	70.56	38.44 – 102.68	**<0.001**	324.71
group [BCS _Meno] × cadherence	1.53	-0.56 – 3.61	0.151	423.69
group [BCS _Ind_Meno] × cadherence	0.72	-1.18 – 2.61	0.457	450.69
time [post] × cadherence	0.54	-0.22 – 1.29	0.165	345.03
cage × cadherence	-0.04	-0.10 – 0.03	0.271	349.89
cbmi × cadherence	-0.01	-0.12 – 0.10	0.821	381.18
hormonal therapy [2] × cadherence	-1.44	-3.53 – 0.64	0.174	358.83
hormonal therapy [4] × cadherence	-0.78	-2.57 – 1.01	0.393	342.03
radiotherapy [2] × cadherence	-0.13	-1.68 – 1.41	0.865	341.03
chemotherapy [2] × cadherence	-1.05	-2.66 – 0.57	0.203	341.19
hypertension therapy [1] × cadherence	-0.40	-1.29 – 0.49	0.381	341.05
csleep × cadherence	-0.28	-0.60 – 0.04	0.090	663.42
cmets × cadherence	-0.03	-0.45 – 0.39	0.892	659.36
group [BCS _Meno] × time [post]) × cadherence	0.71	-0.64 – 2.06	0.299	329.41

group [BCS_ind_meno] × time [post]) × cadherence	-0.49	-1.86 – 0.88	0.480	327.47
**Random Effects**				
σ^2	5707.57			
$\tau_{00\ id}$	1903.38			
ICC	0.25			
N $_{id}$	316			
Observations	632			
Marginal R^2 / Conditional R^2	0.675 / 0.756			

Notes: NEPA, non-exercise physical activity; cage, centered age; cbmi, centered BMI; hormonal therapy [2], ARO_inhib_AnalogGnRH; hormonal therapy [3], Antiestrog_AnalogGnRH; hormonal therapy [4], Antiestrogen; hormonal therapy [no], Non-oncologic women; radiotherapy [2], Breast Cancer survival who had radiotherapy; radiotherapy [no], Non-oncologic women; chemotherapy [2], Breast Cancer survival who had chemotherapy; hypertension therapy [1], taking drugs for hypertension; csleep, centered sleep; cmets, centered Metabolic equivalent of task; cadherence, centered adherence; BCS_Meno, Breast Cancer survival with natural menopause; BCS_Ind_Meno, Breast Cancer survival with medically induced menopause; σ2, residual variance; τ00 id, random intercept for subjects; ICC, interclass correlation coefficient; N id, number of subjects.

Supplementary Table S4. Linear mixed-effect model for sleep.

	**Sleep**			
Predictors	*Estimates*	*CI*	*p*	*df*
(Intercept)	376.72	366.20 – 387.23	**<0.001**	589.98
group [BCS_Meno]	26.52	10.05 – 42.98	**0.002**	589.98
group [BCS_Ind_Meno]	25.66	6.73 – 44.60	**0.008**	589.98
time [post]	17.39	4.79 – 29.98	**0.007**	319.03
group [BCS_Meno] × time [post]	-4.25	-23.97 – 15.47	0.672	319.03
group [BCS_Ind_Meno] × time [post]	0.76	-21.92 – 23.44	0.947	319.03
**Random Effects**				
σ^2	3003.80			

$\tau_{00\ id}$	1200.14
ICC	0.29
N id	316
Observations	632
Marginal R^2 / Conditional R^2	0.050 / 0.322

Notes: BCS_Meno, Breast Cancer survival with natural menopause; BCS_Ind_Meno, Breast Cancer survival with medically induced menopause; σ2, residual variance; τ00 id, random intercept for subjects; ICC, interclass correlation coefficient; N id, number of subjects.

Supplementary Table S5. ANCOVA for SED.

	SED			
Predictors	*Estimates*	*CI*	*p*	*df*
(Intercept)	236.94	105.15 – 368.72	**<0.001**	304.56
group [BCS_Meno]	51.60	-75.38 – 178.57	0.425	309.88
group [BCS_Ind_Meno]	40.77	-90.90 – 172.45	0.543	311.26
time [post]	11.98	-8.76 – 32.72	0.257	314.19
cadherence	0.27	-0.33 – 0.86	0.376	607.49
SED T_0	0.47	0.42 – 0.52	**<0.001**	368.41
cage	0.04	-1.18 – 1.26	0.946	299.29
cbmi	-3.49	-5.28 – -1.70	**<0.001**	326.94
training group	10.33	-4.59 – 25.26	0.174	304.65
hormonal therapy [2]	-40.75	-98.80 – 17.31	0.168	301.75
hormonal therapy [4]	-27.52	-54.71 – -0.33	**0.047**	302.64
hormonal therapy [no]	32.52	-91.90 – 156.94	0.607	298.65
radiotherapy [2]	-1.70	-25.27 – 21.88	0.888	298.62
chemotherapy [2]	15.31	-9.70 – 40.31	0.229	299.70
tr hypertension [1]	-11.86	-28.06 – 4.34	0.151	303.83
csleep	-32.42	-39.92 – -24.92	**<0.001**	586.08
cmets	-78.38	-88.23 – -68.53	**<0.001**	585.59
group [BCS_Meno] × time [post]	-29.98	-62.59 – 2.63	0.071	308.89

group [BCS_Ind_Meno] × time [post]	-73.24	-109.94 – -36.55	**<0.001**	309.39
group [BCS_Meno] × cadherence	-0.34	-1.43 – 0.75	0.538	607.53
group [BCS_Ind_Meno] × cadherence	-0.41	-1.52 – 0.70	0.468	607.45
time [post] × cadherence	-0.12	-0.96 – 0.71	0.771	308.86
(group [BCS_Meno] × time [post]) × cadherence	-0.39	-1.92 – 1.14	0.617	310.05
(group [BCS_Ind_Meno] × time [post]) × cadherence	0.44	-1.12 – 2.01	0.576	309.16
**Random Effects**				
σ^2	7661.55			
$\tau_{00\ id}$	0.00			
N_{id}	316			
Observations	632			
Marginal R^2 / Conditional R^2	0.705 / NA			

Notes: SED, sedentary behavior; cage, centered age; cbmi, centered BMI; hormonal therapy [2], ARO_inhib_AnalogGnRH; hormonal therapy [3], Antiestrog_AnalogGnRH; hormonal therapy [4], Antiestrogen; hormonal therapy [no], Non-oncologic women; radiotherapy [2], Breast Cancer survival who had radiotherapy; radiotherapy [no], Non-oncologic women; chemotherapy [2], Breast Cancer survival who had chemotherapy; hypertension therapy [1], taking drugs for hypertension; csleep, centered sleep; cmets, centered Metabolic equivalent of task; cadherence, centered adherence; BCS_Meno, Breast Cancer survival with natural menopause; BCS_Ind_Meno, Breast Cancer survival with medically induced menopause; σ2, residual variance; τ00 id, random intercept for subjects; N id, number of subjects.

Supplementary Table S6. ANCOVA for NEPA.

	NEPA			
Predictors	*Estimates*	*CI*	*p*	*df*
(Intercept)	292.93	176.62 – 409.24	**<0.001**	307.12
Group [BCS_Meno]	-59.50	-171.76 – 52.77	0.298	309.66
group [BCS_Ind_Meno]	-61.94	-178.39 – 54.50	0.296	311.06
time [post]	-38.11	-56.42 – -19.81	**<0.001**	314.00
cadherence	-0.48	-1.00 – 0.05	0.075	607.58

NEPA T_0	0.39	0.34 – 0.45	**<0.001**	389.56
cage	-0.08	-1.16 – 1.00	0.883	299.46
cbmi	2.68	1.10 – 4.26	**0.001**	327.81
training group	-4.72	-17.88 – 8.44	0.481	303.31
hormonal therapy [2]	11.13	-40.00 – 62.26	0.669	303.95
hormonal therapy [4]	15.52	-8.50 – 39.55	0.205	302.32
hormonal therapy [no]	-34.87	-144.77 – 75.02	0.533	298.86
radiotherapy [2]	4.12	-16.70 – 24.94	0.697	298.76
chemotherapy [2]	-18.00	-40.08 – 4.09	0.110	300.29
tr hypertension [1]	0.13	-14.18 – 14.44	0.986	303.75
csleep	-4.18	-10.68 – 2.33	0.208	574.51
cmets	86.26	77.16 – 95.36	**<0.001**	596.50
group [BCS_Meno] × time [post]	12.66	-16.14 – 41.47	0.388	308.86
group [BCS_Ind_Meno] × time [post]	77.33	44.91 – 109.75	**<0.001**	309.36
group [BCS_Meno] × cadherence	0.42	-0.54 – 1.38	0.387	607.54
group [BCS_Ind_Meno] × cadherence	0.48	-0.50 – 1.46	0.336	607.46
time [post] × cadherence	0.30	-0.43 – 1.04	0.416	308.83
(group [BCS_Meno] × time [post]) × cadherence	1.17	-0.18 – 2.53	0.089	310.08
(group [BCS_Ind_Meno] × time [post]) × cadherence	-0.31	-1.69 – 1.07	0.656	309.14
**Random Effects**				
σ^2	5978.35			
$\tau_{00\ id}$	0.00			
N id	316			
Observations	632			
Marginal R^2 / Conditional R^2	0.748 / NA			

Notes: NEPA, non-exercise physical activity; cage, centered age; cbmi, centered BMI; hormonal therapy [2], ARO_inhib_AnalogGnRH; hormonal therapy [3], Antiestrog_AnalogGnRH; hormonal therapy [4], Antiestrogen; hormonal therapy [no], Non-oncologic women; radiotherapy [2], Breast Cancer survival who had radiotherapy; radiotherapy [no], Non-oncologic women; chemotherapy [2], Breast Cancer survival who had chemotherapy; hypertension therapy [1], taking drugs for hypertension; csleep, centered sleep; cmets, centered Metabolic equivalent of task; cadherence, centered adherence; BCS_Meno, Breast Cancer survival with natural menopause; BCS_Ind_Meno, Breast Cancer survival with medically induced menopause; σ2, residual variance; τ00 id, random intercept for subjects; N id, number of subjects.

Supplementary Table S7. ANCOVA for sleep.

	**Sleep**			
Predictors	*Estimates*	*CI*	*p*	*df*
(Intercept)	140.24	117.83 – 162.64	**<0.001**	353.02
group [BCS_Meno]	9.87	-2.54 – 22.29	0.119	624.85
group [BCS_Ind_Meno]	9.55	-4.70 – 23.80	0.188	624.92
time [post]	17.39	6.23 – 28.54	**0.002**	313.00
sleep baseline	0.63	0.57 – 0.68	**<0.001**	312.00
group [BCS_Meno] × time [post]	-4.25	-21.71 – 13.22	0.633	313.00
group [BCS_Ind_Meno] × time [post]	0.76	-19.33 – 20.85	0.940	313.00
**Random Effects**				
σ^2	2379.19			
$\tau_{00\ id}$	0.00			
N_{id}	316			
Observations	632			
Marginal R^2 / Conditional R^2	0.466 / NA			

Notes: BCS_Meno, Breast Cancer survival with natural menopause; BCS_Ind_Meno, Breast Cancer survival with medically induced menopause; σ2, residual variance; τ00 id, random intercept for subjects; N id, number of subjects.

Supplementary Table S8. Multicollinearity diagnostics: variance inflation factors (VIFs) for LME SED and NEPA models.

		SED				
Correlation Level	*Term*	*VIF*	*VIF 95% CI*	*adj. VIF*	*Tolerance*	*Tolerance 95% CI*
Low	cage	2.36	[2.11, 2.67]	1.54	0.42	[0.37, 0.47]
Low	cbmi	1.59	[1.45, 1.77]	1.26	0.63	[0.56, 0.69]
Low	chemotherapy	1.50	[1.37, 1.67]	1.22	0.67	[0.60, 0.73]
Low	tr_hypertension	1.37	[1.26, 1.52]	1.17	0.73	[0.66, 0.79]
Low	csleep	1.14	[1.07, 1.27]	1.07	0.88	[0.79, 0.94]
Low	cmets	1.57	[1.43, 1.75]	1.25	0.64	[0.57, 0.70]
Low	time	2.30	[2.06, 2.60]	1.52	0.43	[0.39, 0.49]
Low	cadherence	4.64	[4.08, 5.30]	2.15	0.22	[0.19, 0.25]
Low	Group:time	4.81	[4.22, 5.50]	2.19	0.21	[0.18, 0.24]
Low	time:cadherence	3.27	[2.90, 3.72]	1.81	0.31	[0.27, 0.35]

Low	cage:cadherence	2.14	[1.92, 2.40]	1.46	0.47	[0.42, 0.52]
Low	cbmi:cadherence	2.17	[1.94, 2.44]	1.47	0.46	[0.41, 0.51]
Low	hormonal_therapy:cadherence	4.09	[3.60, 4.67]	2.02	0.24	[0.21, 0.28]
Low	chemotherapy:cadherence	2.01	[1.81, 2.26]	1.42	0.50	[0.44, 0.55]
Low	tr_hypertension:cadherence	2.52	[2.25, 2.85]	1.59	0.40	[0.35, 0.44]
Low	csleep:cadherence	1.26	[1.17, 1.40]	1.12	0.79	[0.71, 0.86]
Low	cmets:cadherence	1.57	[1.43, 1.75]	1.25	0.64	[0.57, 0.70]
Moderate	radiotherapy:cadherence	7.26	[6.34, 8.34]	2.69	0.14	[0.12, 0.16]
Moderate	Group:time:cadherence	5.14	[4.51, 5.89]	2.27	0.19	[0.17, 0.22]
High	hormonal_therapy	281.38	[242.83, 326.09]	16.77	0.00355	[0.00, 0.00]
High	radiotherapy	102.67	[88.65, 118.93]	10.13	0.00974	[0.01, 0.01]

High	Group:cadherence	37.97	[32.83, 43.94]	6.16	0.03	[0.02, 0.03

NEPA

Correlation Level	Term	VIF	VIF 95% CI	adj. VIF	Tolerance	Tolerance 95% CI
Low	cage	2.36	[2.12, 2.67]	1.54	0.42	[0.37, 0.47]
Low	cbmi	1.60	[1.45, 1.78]	1.26	0.63	[0.56, 0.69]
Low	chemotherapy	1.50	[1.37, 1.67]	1.22	0.67	[0.60, 0.73]
Low	tr_hypertension	1.37	[1.26, 1.52]	1.17	0.73	[0.66, 0.79]
Low	csleep	1.14	[1.07, 1.27]	1.07	0.88	[0.78, 0.93]
Low	cmets	1.59	[1.45, 1.77]	1.26	0.63	[0.56, 0.69]
Low	time	2.30	[2.06, 2.60]	1.52	0.43	[0.39, 0.49]
Low	cadherence	4.74	[4.16, 5.42]	2.18	0.21	[0.18, 0.24]
Low	time:cadherence	3.36	[2.97, 3.82]	1.83	0.30	[0.26, 0.34]
Low	cage:cadherence	2.14	[1.92, 2.41]	1.46	0.47	[0.42, 0.52]

Low	cbmi:cadherence	2.18	[1.95, 2.45]	1.48	0.46	[0.41, 0.51]
Low	hormonal_therapy:cadherence	4.10	[3.61, 4.68]	2.02	0.24	[0.21, 0.28]
Low	chemotherapy:cadherence	2.01	[1.81, 2.26]	1.42	0.50	[0.44, 0.55]
Low	tr_hypertension:cadherence	2.52	[2.25, 2.85]	1.59	0.40	[0.35, 0.44]
Low	csleep:cadherence	1.26	[1.17, 1.40]	1.12	0.79	[0.71, 0.85]
Low	cmets:cadherence	1.58	[1.44, 1.77]	1.26	0.63	[0.57, 0.69]
Moderate	Group:time	5.03	[4.41, 5.76]	2.24	0.20	[0.17, 0.23]
Moderate	radiotherapy:cadherence	7.26	[6.34, 8.34]	2.69	0.14	[0.12, 0.16]
Moderate	Group:time:cadherence	5.43	[4.76, 6.22]	2.33	0.18	[0.16, 0.21]
High	hormonal_therapy	287.25	[247.89, 332.89]	16.95	0.00348	[0.00, 0.00]
High	radiotherapy	102.77	[88.74, 119.06]	10.14	0.00973	[0.01, 0.01]
High	Group:cadherence	38.83	[33.57, 44.94]	6.23	0.03	[0.02, 0.03]

Note: SED, sedentary behavior; NEPA, non-exercise physical activity; cage, centered age; cBMI, centered BMI; csleep, centered sleep; cmets, centered Metabolic Equivalent of Task; cadherence, centered adherence.

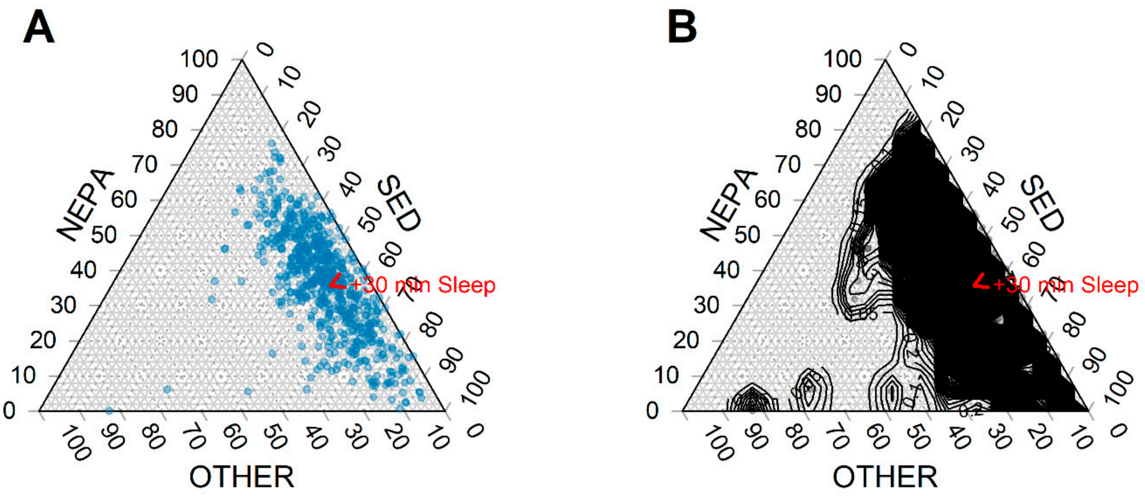

Supplementary Figure S1. Wake-time composition of NEPA, SED, and OTHER behaviors represented in a ternary plot. Each point corresponds to a day, normalized to the wake-time subcomposition (NEPA + SED + OTHER = 1). Panel A shows individual observations (scatter), while Panel B overlays kernel density contours to highlight the most frequent wake-time patterns. The red arrow illustrates the compositional shift when + 30 minutes are reallocated to sleep by proportionally reducing SED and NEPA.
